# Good news for sex workers in Zimbabwe: how a court order improved safety in the absence of decriminalization

**DOI:** 10.7448/IAS.20.1.21860

**Published:** 2017-05-15

**Authors:** Joanna Busza, Sibongile Mtetwa, Elizabeth Fearon, David Hofisi, Tinashe Mundawarara, Raymond Yekeye, Tapuwa Magure, Owen Mugurungi, Frances Cowan

**Affiliations:** ^a^ Department of Population Health, London School of Hygiene & Tropical Medicine, London, UK; ^b^ Centre for Sexual Health and HIV/AIDS Research, Harare, Zimbabwe; ^c^ Department of Social and Environmental Health Research, London School of Hygiene & Tropical Medicine, London, UK; ^d^ Zimbabwe Lawyers for Human Rights, Harare, Zimbabwe; ^e^ Zimbabwe National AIDS Council, Harare, Zimbabwe; ^f^ TB and AIDS Unit, Zimbabwe Ministry of Health, Harare, Zimbabwe; ^g^ Department of International Public Health, Liverpool School of Tropical Medicine, Liverpool, UK

As highlighted in the *JIAS* special issue on the role of police in the global HIV pandemic, legal policy and its enforcement both reflect and shape social attitudes toward key populations including sex workers, men who have sex with men, drug users and other marginalized groups [1]. How the police interpret and implement legal restrictions on specific behaviours influences local risk environments by sharpening or mitigating the laws’ impact [2–4]. This article summarizes recent legal action in Zimbabwe that has positively influenced female sex workers’ (FSW) safety and, if sustained, is likely to translate into reduced HIV risk.

Criminalization of sex work exacerbates FSW’s vulnerability to HIV and other adverse health outcomes [5,6]. Sex workers’ use of HIV prevention and treatment services are lower among those reporting arrest or police harassment in a wide range of settings [4,7–9]. Fear of police also increases FSW’s risk-taking with clients as they have less time to negotiate condom use or assess safety prior to transacting sex [3,10]. The preponderance of evidence linking criminalization of sex work to HIV risk has led to repeated calls for decriminalization [11–14], yet selling sex remains illegal in over 100 countries, with associated laws against solicitation, loitering, antisocial behaviour or bringing a country’s morals into disrepute further used to control and abuse sex workers [15,16].

Policy reform or reinterpretation can lead to positive change. The *JIAS* special issue made a point of focusing on positive examples, including how changes in legal status in New Zealand led to improvements in women’s safety and their ability to rely on police to address problems with clients [17]. Other examples include reduction in arrests and police abuse of FSW in India following advocacy by community-based organizations representing sex workers’ rights [18,19] and a pilot project in Durban, South Africa, that brought together FSW and police in sensitization workshops [20].

In Zimbabwe, the burden of HIV among FSW is exceedingly high, at over 50% [21]. The Centre for Sexual Health, HIV and AIDS Research (CeSHHAR) has conducted multiple respondent-driven sampling (RDS) surveys among self-identified sex workers, and has measured rates of reported police harassment and abuse, with up to 20% FSW reporting violence from the police in the past year [22]. Our qualitative research has further documented the way in which fear of arrest endangers women working in bars, truck stops and the street by making it unlikely women will seek redress if they experience violence, and reducing their willingness to engage in long negotiations in situations where they might attract police attention [23].

A recent court order, however, appears to have resulted in significant change in relations between sex workers and the police. In 2014, nine women from Harare were arrested for solicitation and subsequently convicted. The law stipulates, however, that both the *conduct* of solicitation must be specified (i.e. evidence of proactive attempt to procure a client rather than based on the person’s location and/or clothing) and the person *who was being solicited* must be present in court. Based on the absence of these requirements, Zimbabwe Lawyers for Human Rights (ZLHR) took the case to the Constitutional Court, successfully arguing that the women’s conviction was in violation of the newly ratified 2013 Constitution’s Sections 49(1)(b) and 56(1), respectively:

- Right to Personal Liberty … which includes the right …not to be deprived of their liberty arbitrarily or without just cause
- All persons are equal before the law and have the right to equal opportunities in political, economic, cultural and social spheres

A court order in favour of the sex workers’ case was issued in June 2015 and widely reported in Zimbabwe’s media as signifying that the police were no longer allowed to arrest sex workers [24–26]. The country’s first lady also referred to the case, implying sex work had been decriminalized [27]. Since the court order, the number of FSW arrests reported to ZLHR has dropped to the extent that they have not yet been able to find a new case with which to test the order.

We analysed our RDS data pooled across 14 sites for two survey rounds, December 2013 and March 2016. At baseline, 50.4% of 2722 FSW reported having been stopped by the police in the past year but by 2016 this had dropped to 29.6% of 2883 FSW (RDS-II weighted [28], unpublished data). While the survey was not designed to monitor changes in policing, it does appear to confirm anecdotal reports from across Zimbabwe.

At a staff meeting, we spoke to CeSHHAR peer educators about the change, who observed the following:

We went into the streets to celebrate the freedom of movement granted to us!

We used to be rounded up in the streets even if we were caught just standing there, but now they can’t do that unless there is a client there as well. So cops are finding it hard to arrest us. I’m sure it will also make them think and realise that we are also human beings.

To discuss issues to do with SW will be well received by policymakers, as they all know the decision that was made in court in our favour.

Data from these sources thus suggest the recent legal reinterpretation of solicitation law has led to change in police practice and police harassment is no longer one of the primary challenges faced by street-based FSW. Whether improvements in work conditions will be sustained and translate into risk reduction for sex workers remains to be seen.

As previously documented, negative political and economic dynamics contributed to Zimbabwe’s HIV epidemic [29]. We thus feel it is important to share this positive example of a successful structural intervention in a difficult context. Even in the absence of full decriminalization, legal measures can affect determinants of HIV prevention and treatment in a relatively short period of time. This adds to the growing body of literature providing positive models for legal and judicial measures conducive to protecting and promoting the health and rights of key populations.
